# Predicting lithium treatment response in bipolar patients using gender-specific gene expression biomarkers and machine learning

**DOI:** 10.12688/f1000research.14451.3

**Published:** 2018-12-07

**Authors:** Andy R. Eugene, Jolanta Masiak, Beata Eugene

**Affiliations:** 1 Independent Researcher, Kansas, USA; 2 Department of Pharmacogenomics, Bernard J. Dunn School of Pharmacy, Inova Center for Personalized Health, Shenandoah University, Fairfax, VA, 22031, USA; 3 Independent Neurophysiology Laboratory, Department of Psychiatry, Medical University of Lublin, Lublin, 20-439, Poland; 4 Marie-Curie Sklodowska University, Lublin, 20-400, Poland

**Keywords:** lithium, treatment response, gene expression, machine learning, microarray, transcriptome, precision medicine, pharmacogenomics, psychiatry, genomic medicine

## Abstract

**Background: **We sought to test the hypothesis that transcriptome-level gene signatures are differentially expressed between male and female bipolar patients, prior to lithium treatment, in a patient cohort who later were clinically classified as lithium treatment responders.

**Methods: **Gene expression study data was obtained from the Lithium Treatment-Moderate dose Use Study data accessed from the National Center for Biotechnology Information’s Gene Expression Omnibus via accession number GSE4548. Differential gene expression analysis was conducted using the Linear Models for Microarray and RNA-Seq (limma) package and the Decision Tree and Random Forest machine learning algorithms in R.

**Results: **Using quantitative gene expression values reported from patient blood samples, the RBPMS2 and LILRA5 genes classify male lithium responders with an area under the receiver operator characteristic curve (AUROC) of 0.92 and the ABRACL, FHL3, and NBPF14  genes classify female lithium responders AUROC of 1. A Decision Tree rule for establishing male versus female samples, using gene expression values were found to be: if RPS4Y1 ≥ 9.643, patient is a male and if RPS4Y1 < 9.643, patient is female with a probability=100%.

**Conclusions:** We developed a pre-treatment gender- and gene-expression-based predictive model selective for classifying male lithium responders with a sensitivity of 96% using 2-genes and female lithium responders with sensitivity=92% using 3-genes.

## Introduction

Lithium is the most well-established mood-stabilizer in the practice of psychiatry ( Jermain *et al*., 1991; Landersdorfer *et al*., 2017). A recent propensity-score adjusted and matched longitudinal cohort-study evaluating the effectiveness of the newer mood stabilizers: olanzapine (n=1477), quetiapine (n=1376), and valproate (n=1670), in comparison to lithium (n=2148), found that patients treated with lithium experienced reduced rates of both unintentional injury and self-harm ( Hayes *et al*., 2016). However, due to lithium’s narrow index of 0.5–1.2 mEq/mL, Therapeutic Drug Monitoring (TDM) is the standard-of-care to ensure patient safety using pharmacokinetic principles in medical practice ( Hiemke *et al*., 2011). Actually, if TDM is applied broadly among medical specialties, pharmacogenomic reports that focus on pharmacokinetic-based gene-drug interactions (e.g. CYP2D6-Paroxetine or CYP2C19-Clopidogrel) may not be necessary in all cases and insurance reimbursement would not be a rate-limiting step in advancing genomic medicine. Although, this approach alone would not account for the hypersensitivity-type pharmacogenomic reactions; however, a TDM pharmacogenomic-hypersensitivity reaction hybrid approach may be an option when concerns about the electronic medical record costs, genotyping and/or sequencing machine costs, and data server infrastructure costs are prohibitive factors causing hospital systems and primary care clinics not to implement pharmacogenomic testing.

A limitation of TDM-only approach, rather than a gene-drug testing, is that one would need to administer the drug and measure a blood concentration after the drug is administered, which may not be an option in life-threatening cases (e.g. stent thrombosis and Clopidogrel). Contrastingly, a profound area of concern for pharmacogenomic testing reports are that hospitals are not implementing actionable pharmacogenomic alerts in the patient medical records if the patient did not have the pharmacogenomic testing at their hospital laboratory due concerns of being a certified genomics laboratory and concerns of litigation when knowingly prescribing a drug that the patient cannot metabolize and scanned into the medical record.

It is important to note that pharmacogenomic reports do not necessarily account for drug-drug-gene interactions – which are often the case – when patients are prescribed three or more medications. In such cases, hospital systems should embed clinical pharmacologist physicians, as is done by leading hospitals globally (e.g. Karolinska Institute in Stockholm Sweden awarding the Nobel Prize, the Mayo Clinic, and more) that aim to maintain high rates of patient drug safety and hospital quality outcome measures ( Eichelbaum *et al*., 2018; Eugene & Eugene, 2018). However, even after accounting for drug doses and drug selection to avoid adverse drug reactions, divergent clinical response rates, among genders, are well-known and reported in psychiatric patients treated with lithium ( Viguera *et al*., 2000).

In a 1986, Zetin and colleagues published the results of a study that evaluated four methods for predicting lithium daily dosages, and the final equation resulted in a 147.8mg/day increased dosage-adjustment for male patients ( Zetin *et al*., 1986). Similarly, a later study by Lobeck and colleagues corroborated the 147.8 mg/day male increase dose requirement for the lithium maintenance dose in bipolar patients ( Lobeck *et al*., 1987). However, neither do the current dosing guidelines recommend a gender-based dose adjustment using pharmacometrics methods, to avoid toxicity, nor are gender-specific gene expression screening panels available to predict lithium efficacy currently available and implemented.

A recent large-scale meta-analysis of human body-tissue gene expression reported that the body organ with the most abundant gender-biased gene expression is the anterior cingulate cortex within the frontal cortex of the brain ( Mayne *et al*., 2016). Thus, these findings suggest that therapeutic drug response may be influenced not only via drug absorption, distribution, metabolism, and elimination, but also within the underlying gene signatures across the human transcriptome and mechanisms of gene-gene interactions that regulate physiology. Beech and colleagues conducted a study to identify gene expression differences from the peripheral blood in patients classified as lithium responders and non-responders ( Beech *et al*., 2014). However, the study reported that no significant gender-biased gene expression differences were found (p-value=0.941) in patients who were randomized to optimal therapy (control), defined as one FDA-approved mood stabilizer, versus patients treated with lithium plus optimal therapy ( Beech *et al*., 2014). Despite these initially reported findings, a recent study by Labonté and colleagues, which used RNA-Seq to evaluate the transcriptome in patients diagnosed with major depressive disorder (MDD), concluded that gender dimorphism exists at the transcriptome-level in MDD patients and that gender-specific treatments should be investigated ( Labonté *et al*., 2017).

Therefore, there is a clinical need to investigate if indeed a gender dimorphism exists in lithium treatment by applying a combination of statistics and data science/engineering methods to advance precision and genomic medicine in psychiatry. These findings may improve prediction of clinical drug response of lithium prior to initiating drug therapy in patients with bipolar or schizoaffective disorders, who often cannot risk drug inefficacy for obvious safety reasons. Therefore, the overall aim for our study is to define gender-specific transcriptional-level regulators of lithium treatment response that may influence treatment of bipolar or schizoaffective disorders. We will test the hypothesis that biologically plausible gene expression differences exist, prior to lithium treatment, in patients diagnosed with bipolar disorder in the following three patient subgroups: (1) male and female patients who were later clinically classified as lithium treatment responders; (2) male-responders versus male-non-responders; (3) female-responders versus female-non-responders.

## Methods

### Data

DNA microarray data analyzed in this study are originally referenced from the Lithium Treatment-Moderate dose Use Study placed in the National Center for Biotechnology Information (NCBI) Gene Expression Omnibus (GEO) via accession number GSE45484 with the Illumina HumanHT12 V4.0 expression Beadchip GPL10558 platform file to associate gene names and descriptions. The original multisite clinical study recruited patients from Case Western Reserve University, Massachusetts General Hospital, Stanford University, Yale University, and the Universities of: Pittsburgh, Texas Health Science Center at San Antonio, and Pennsylvania ( Beech *et al*., 2014). From the original 120 peripheral blood samples used to generate probe and gene expression profiles, from patients diagnosed with bipolar disorder, the clinical phenotype of being either a treatment- responder or non-responder was assessed using the Clinical Global Impression Scale for Bipolar Disorder-Severity (CGI-BP-S) ( Spearing *et al*., 1997).

### Study design

To assess for gender-specific differential gene signatures, in our first analysis we grouped patients based on gender alone and not on any other variables (i.e. optimal treatment versus lithium, or responder versus non-responder status). Then, we rationalized that from the results of the gender-specific transcriptome signatures from our first analysis, we will set the top two-hundred and fifty genes as controls in an effort to identify pharmacologic treatment-response transcriptome biomarkers that are not directly linked to the X or Y chromosome. Therefore, we *overlaid* the top two-hundred and fifty genes from all results that were reported in subsequent analyses to identify genes with lithium-specific transcriptional differences between genders associated with response to Lithium treatment. In our second analysis, we only selected patients who were classified as lithium treatment-responders, at baseline, and the results from the gene expression differences are reported excluding the sex-specific control genes identified in the first experiment. In our third and fourth analyses, we compared: male-responders vs. male non-responders, and female-responders vs. female non-responders, respectively.

### Machine learning

**Figure 1.**
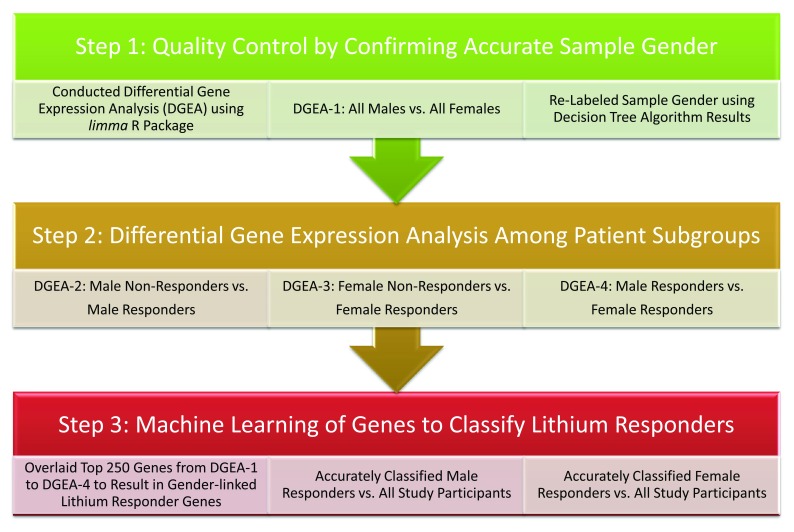
Data analysis workflow used to accurately classify and label sample-gender and gender-specific lithium treatment responders. Heatmaps were created following identification of the top differentially expressed genes and Variable Importance plots were produced following identification of gender-specific lithium treatment responders.

A graphical depiction of the data analysis methods are shown in Figure 1. The *Decision Tree* and *Random Forest* machine learning algorithms were used for classification following identification of statistically significant DNA microarray genes. This method sets the stage for subsequent analyses aiming to identify gender-specific responder genes with small sample size of three male-responders and six-female responders from the total of sixty patients. Thus, to reiterate, we first utilized the significant results obtained from the gene expression package implemented in the *limma* package in R and then applied the *Decision Tree* and *Random Forest* algorithms for classification and determined this to be novel.

To identify if patients were either male or female, we divided the dataset of 120 samples, pre-treatment and post-treatment, from sixty patients into three sub-datasets: (1) training dataset (60% of total sample), (2) validation dataset (20% of total sample), and the (3) test dataset (20% of total sample). However, due to having small lithium treatment responder sample-sizes, when identifying gender-specific responders versus ‘All Other Patients’, we simply used a training dataset (70% of total) and a test dataset (30% of total sample). We then reported the classification performance of the models using the following diagnostic parameters: sensitivity, specificity (not calculated for gender-specific lithium responders due sample size), and an area under the receiver operator characteristic curve (AUROC). We selected the traditional Decision Tree algorithm to classify male versus female samples using the following parameters: complexity of 0.01, a max depth of 3, minimum bucket of 7, and a minimum split of 20 observations. Further, for classifying male-responders and female-responders, we selected the *Random Forest* algorithm and set the number of Trees to build at 500 with 7 variables at any time for dataset partitioning. Finally, we reported variable importance plots of genes throughout the paper that was used to explain which genes were most important for classifying patients into different reportable subgroups. Final results of the *Random Forest* processes for male- and female-responders are located in Supplementary File 1.

### Gene expression analysis

Differential gene expression analysis of the DNA microarray data was conducted using the Empirical Bayes method implemented within the *limma* package (version 3.34.5) and utilizes the Biobase package (version 2.38.0) which both run within the R for Statistical Programming environment (version 3.4.3; R Foundation for Statistical Computing, Vienna, Austria) ( Ritchie *et al*., 2015; Team, 2013). Due to multiple testing of the peripheral blood transcriptome, the False-Discovery Rate was adjusted using the Benjamini-Hochberg method. A p-value of less 0.05 was considered to be statistically significant and a differential gene expression threshold of 0.5 was used and reported during the machine learning process.

## Results

**Figure 2.**
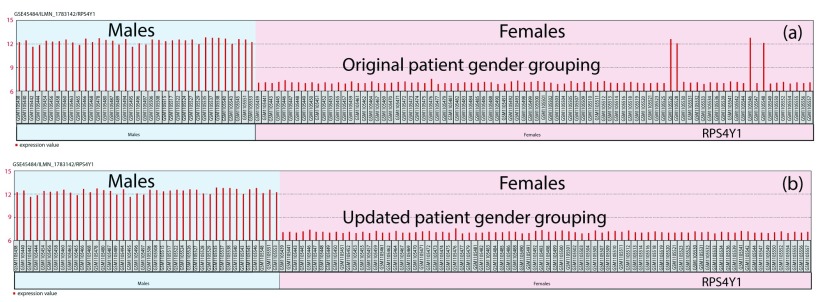
Gene expression levels for the Ribosomal protein S4, Y-linked 1 (RPS4Y1) gene illustrating 4 patient samples as labeled as female and were re-assigned to the male patient gender group.

**Figure 3.**
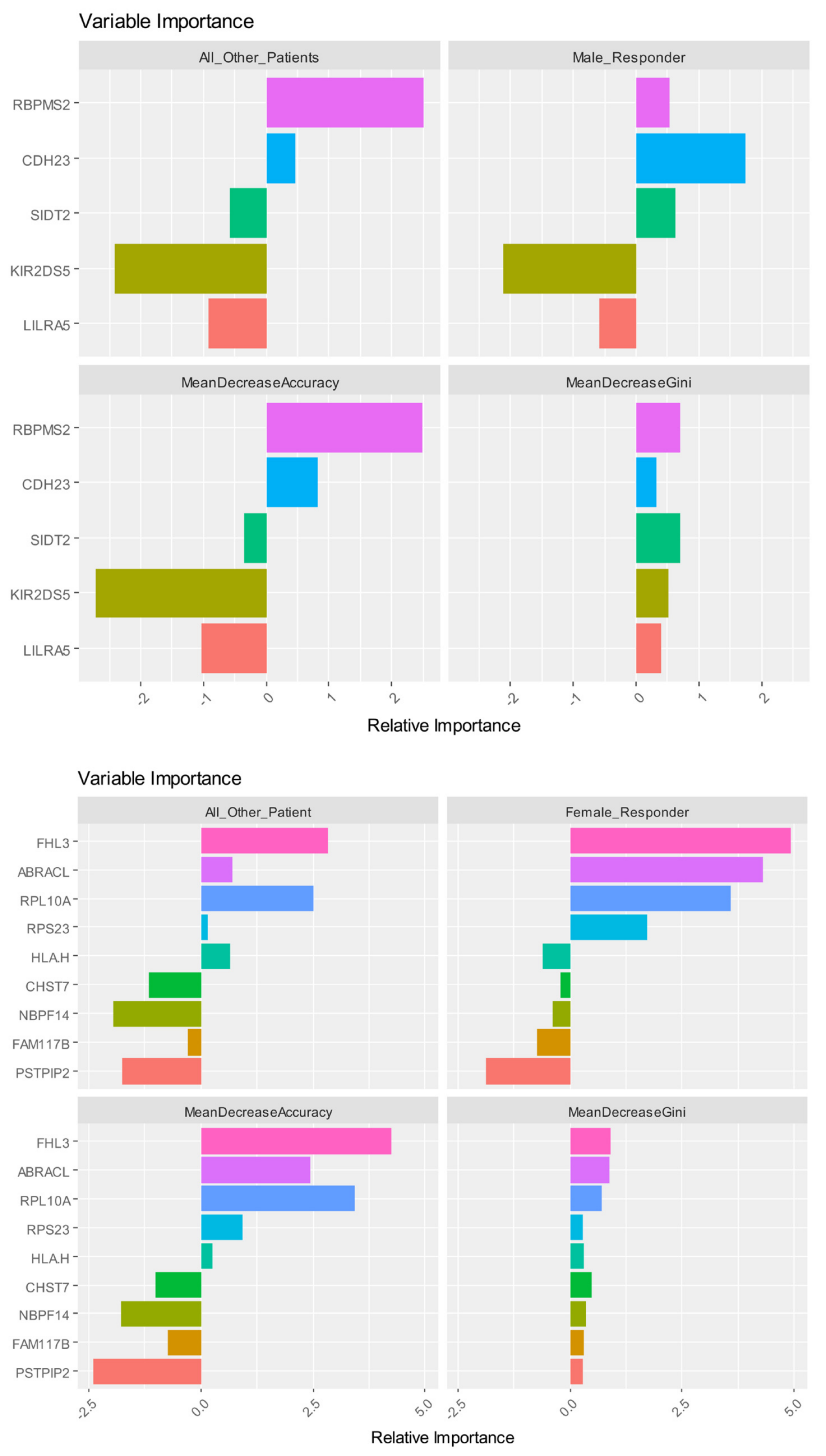
Variable importance ratings of genes selective (above) of male lithium responders versus the entire population of treated and untreated patient men and women; and (below) female lithium responders versus the entire population of treated and untreated men and women.

Table 1 provides the patient age and sample sizes used during subgroup analyses. In our first analysis, which aimed to group patients based on gender alone and not based on clinical variables detailed in the original study, data-driven gene analytics identified four female-labeled patient samples with gene expression levels similar to that found in male patients for the following Y-chromosome genes: *RPS4Y1, EIF1AY, KDM5D, RPS4Y2*; and the *XIST* gene located on the X-chromosome. Therefore, all subsequent hypothesis-testing were analyzed with the updated male-gender classification for the following NCBI GEO patient samples: GSM1105526 (baseline lithium-non-responder), GSM1105528 (1-month lithium-non-responder), GSM1105546 (baseline lithium-non-responder), and GSM1105548 (1-month lithium-non-responder). Figure 2 illustrates the gene expression findings resulting in re-classification for the aforementioned patient samples. The Decision Tree rule states: if *RPS4Y1* < 9.643 then the patient is a female with a probability of 100%. Whereas, if the *RPS4Y1* ≥ 9.643 then the patient is a male with a probability of 9%. After proceeding with the machine learning analysis of both the ‘training’ and ‘validation’ datasets, the final ‘test’ dataset resulted in the following diagnostic test evaluation parameters: Sensitivity=100% (95% C.I. 66.37%-100.00%), Specificity=100% (95% C.I. 78.20%-100.00%), and an AUROC of 1. Figure 3 illustrates the variable importance plots used in the machine learning process for classifying patients as being a male-lithium-responder or female-lithium-responder relative to the full patient population. The results show, in descending order of predictive power, the genes selective for male lithium-responders versus the full patient population being RBPMS2, CDH23, and SIDT2. Similarly, in descending order of predictive power, for female lithium-responders versus the entire patient population, the FHL3, ABRACL, RPL10A, and RPS23 genes are most selective.

**Table 1.** Patient age and sample sizes used during subgroup analyses.

Lithium treated patient population			
Baseline	Mean age	S.D.	Sample size (n)
**Male-responder**	36	8.1	3
**Female-responder**	31	11.8	6
**Male-non-responder**	40	10	7
**Female-non-responder**	44	9.2	12
*General mood stabilizers patient population			
Baseline	Mean age	S.D.	Sample size (n)
**Male-responder**	51	--	1
**Female-responder**	49	10.5	3
**Male-non-responder**	43	12.5	9
**Female-non-responder**	37	14.5	19
Total patient population			
Gender	Mean age	S.D.	Sample size (n)
**Male**	41	10.8	20
**Female**	39	13.1	40
**Study population**	40	12.3	60

* **Note:** United States Food and Drug Administration approved Mood Stabilizers.

Table 2 provides the results for the gender-specific differentially expressed genes from the entire study population using a fold-change (FC) threshold of 0.5. A total of five genes met the *a priori* FC requirements and were found to be *RPS4Y1, EIF1AY, KDM5D, RPS4Y2*, and *EIF1AY*. These five down-regulated male-biased genes were all found on the Y-chromosome. Contrastingly, a total of 10 upregulated female-biased genes were found to be: *XIST, S100P, IFIT3, TNFAIP6, IFITM3, IFIT2, CHURC1, ANXA3, ADM*, and *PROK2*. The *RPS4Y1* gene in males (FC= -4.9807, p=7.36E-47) and the *XIST* gene (FC=1.7615, p=2.98E-36), found on the X-chromosome, in females resulted in the greatest expression changes between genders. The male-favored genes resulted in a larger expression change than compared to the females.

**Table 2.** Differentially expressed genes between genders across all study participants with a log fold-change threshold of 0.5.

Male-associated genes					
Gene	Adjusted P-value	P-value	Log fold change	Gene description	Location
**RPS4Y1**	7.36E-47	2.81E-51	-4.9807	Ribosomal Protein S4, Y-linked 1	Yp11.3
**EIF1AY**	1.02E-41	8.61E-46	-2.5861	Eukaryotic Translation Initiation Factor 1A, Y-linked	Yq11.223
**KDM5D**	7.36E-47	4.67E-51	-1.6658	Lysine Demethylase 5D	Yq11
**HLA-DRB1**	0.016	0.0000362	-1.7072	Major Histocompatibility Complex, Class II, DR Beta 1	
**RPS4Y2**	1.35E-40	1.43E-44	-1.5014	Ribosomal Protein S4, Y-linked 2	Yq11.223
**EIF1AY**	9.38E-31	1.98E-34	-0.9443	Eukaryotic Translation Initiation Factor 1A, Y-linked	Yq11.223
Female-associated genes					
Gene	Adjusted P-value	P-value	Log fold change	Gene description	Location
**XIST**	2.98E-36	5.03E-40	1.7615	X Inactive Specific Transcript (non-protein coding)	Xq13.2
**S100P**	1.70E-02	3.31E-05	1.028	S100 Calcium Binding Protein P	4p16
**IFIT3**	5.00E-03	5.82E-06	0.8765	Interferon Induced Protein with Tetratricopeptide Repeats 3	10q24
**TNFAIP6**	3.73E-04	2.52E-07	0.7304	TNF Alpha Induced Protein 6	2q23.3
**IFITM3**	4.51E-02	1.69E-04	0.7284	Interferon Induced Transmembrane Protein 3	11p15.5
**IFIT2**	4.91E-02	1.95E-04	0.6739	Interferon Induced Protein with Tetratricopeptide Repeats 2	10q23.31
**CHURC1**	6.30E-02	3.18E-04	0.6678	Churchill Domain Containing 1	14q23.3
**ANXA3**	2.33E-03	2.26E-06	0.6218	Annexin A3	4q21.21
**ADM**	8.69E-04	6.80E-07	0.5986	Adrenomedullin	11p15.4
**PROK2**	2.16E-02	4.79E-05	0.5189	Prokineticin 2	3p13

**Figure 4.**
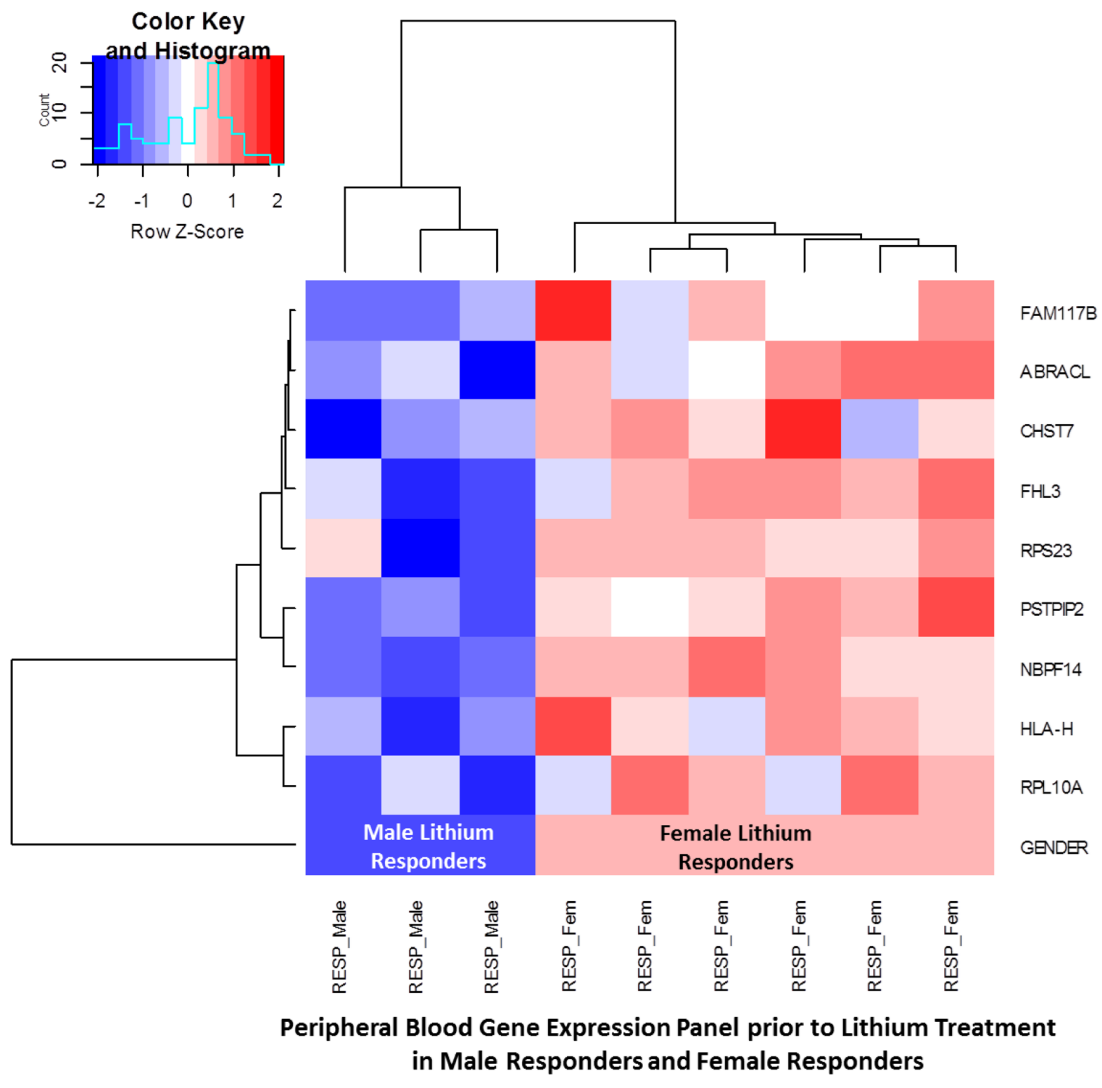
Heat-map and dendrogram overview of the two-way unsupervised hierarchical cluster analysis of differentially expressed genes in male (n=3) and female (n=6) lithium responders after overlaying the top 250 differentially expressed genes found gender biased genes.

Table 3 provides the results for the differentially expressed genes that were found between male and female responders prior to initiation of lithium and optimal therapy, meeting the FC criteria of at least 0.5. In male lithium responders, we found 5 differentially expressed while the RNA binding protein with multiple splicing 2 ( *RBPMS2*) gene ranked with the greatest FC of -1.351 (unadjusted p=0.00111). Whereas, 9 genes were associated with female lithium responders, with greatest expression change being the major histocompatibility complex class-1-H (HLA-H) at 1.602 (unadjusted p-value=0.00099). The neuroblastoma breakpoint family member-14 ( *NBPF14*) gene met the Benjamani-Hochberg adjusted p-value criteria and resulted with an expression change of 0.586 (adjusted p=0.0462). Figure 4 illustrates the heat-map and dendrogram overview of the two-way unsupervised hierarchical cluster analysis of the reported differentially expressed genes among male and female responders to lithium therapy at baseline that correspond to values reported in Table 3.

**Table 3.** Differentially expressed genes between male and female responders prior to Lithium pharmacotherapy with a log fold-change threshold of 0.5.

Genes associated with male lithium responders					
Gene	Adjusted P-value	P-value	Log fold change	Gene description	Highest gene tissue expression
**RBPMS2**	1	0.00111	-1.351	RNA Binding Protein with Multiple Splicing 2	Heart, Urinary Bladder
**SIDT2**	1	0.00932	-0.82	SID1 Transmembrane Family Member 2	Stomach, Prostate
**CDH23**	1	0.00388	-0.674	Cadherin-Related 23	Ovary, Fat
**LILRA5**	1	0.00359	-0.592	Leukocyte Immunoglobulin Like Receptor A5	Appendix, Bone Marrow
**KIR2DS5**	1	0.00431	-0.506	Killer Cell Immunoglobulin Like Receptor, Two Ig Domains and Short Cytoplasmic Tail 5	--
Genes associated with female lithium responders					
Gene	Adjusted P-value	P-value	Log fold change	Gene description	Highest gene tissue expression
**HLA-H**	1	0.000996	1.602	Major Histocompatibility Complex, Class I, H (pseudogene)	Lymph Node, Bone Marrow
**RPS23**	1	0.00308	1.471	Ribosomal Protein S23	Ovary, Bone Marrow
**FHL3**	1	0.000751	0.893	Four and a Half LIM Domains 3	Esophagus, Endometrium
**RPL10A**	1	0.00299	0.628	Ribosomal Protein L10a	Ovary, Bone Marrow
**NBPF14	**0.0462	0.00000782	0.586	Neuroblastoma Breakpoint Family Member 14	Skin, Ovary
**PSTPIP2**	1	0.000473	0.569	Proline-Serine-Threonine Phosphatase Interacting Protein 2	Bone Marrow, Spleen
**FAM117B**	1	0.00949	0.556	Family with Sequence Similarity 117 Member B	Testis, Adrenal
**CHST7**	1	0.00812	0.529	Carbohydrate Sulfotransferase 7	Spleen, Fat
**ABRACL**	1	0.00396	0.505	ABRA C-Terminal Like	Colon, Lymph Node

Notes: **The NBPF14 gene reached the Benjamani-Hochberg adjusted p-value.

Using the baseline blood sample microarray data, the predictive modeling results for identifying lithium-responders from the complete study population of male and female controls and treatment samples, resulted in a validation/test sample cohort for males of: Sensitivity=95.83% (95% C.I. 78.88%-99.89%), Specificity=not calculated due sample size of test dataset, and an AUROC = 0.92 using the *RBPMS2* and *LILRA5* genes. Likewise, in the test dataset for females: Sensitivity=91.67% (95% C.I. 61.52%-99.79%), Specificity= not calculated due sample size of test dataset, and an AUROC = 1 with the *ABRACL*, FHL3, and the *NBPF14* genes. Therefore, we developed a 2-gene predictive model for men and a 3-gene predictive model for women classifying lithium response in bipolar patients from a general population of bipolar patients using transcriptional signatures at baseline, prior to prescribing and treating a patient with lithium.

Table 4 provides the list of 10 differentially expressed genes found in male lithium responders (5-genes) and male lithium-non-responders (5-genes). The RNA binding protein with multiple splicing 2 ( *RBPMS2*) gene (FC= -1.326, unadjusted p=0.001358) in male lithium responders and the Ribosomal protein S23 ( *RPS23*) gene (FC=1.521, unadjusted p=0.013306) were found to result in the largest expression change differences between subgroups. However, in female responders and female non-responders, the Family with Sequence Similarity 117 Member B ( *FAM117B*) gene (FC=0.5257, unadjusted p=0.0048554) and the Golgin B1 ( *GOLGB1*) gene (FC= -0.6536, unadjusted p=0.0003716) were differentially expressed, respectively and shown in Table 5.

**Table 4.** Differentially expressed genes between Male Responders and Male Non-Responders at baseline with a log fold-change threshold of 0.3.

Genes associated with male lithium responders					
Gene	Adjusted P-value	P-value	Log fold change	Gene description	Highest gene tissue expression
**RBPMS2**	1	0.001358	-1.326	RNA Binding Protein with Multiple Splicing 2	Heart, Urinary Bladder
**SVBP**	1	0.01366	-0.76	Small Vasohibin Binding Protein	Testis, Fat
**LILRA5**	1	0.011739	-0.714	Leukocyte Immunoglobulin Like Receptor A5	Appendix, Bone Marrow
**CPA3**	1	0.008048	-0.592	Carboxypeptidase A3	Gall Bladder, Lung
**SLC45A3**	1	0.016508	-0.455	Solute Carrier Family 45 member 3	Prostate, Stomach
**ZNF234**	1	0.003254	-0.41	Zinc Finger Protein 234	Spleen, Thyroid
**DIDO1**	1	0.008232	-0.385	Death Inducer-Obliterator 1	Ovary, Spleen
**TPP2**	1	0.013053	-0.385	Tripeptidyl Peptidase 2	Testis, Thyroid
**KRT73**	1	0.007333	-0.373	Keratin 73	Skin, Lymph Nodes
**ZMYM3**	1	0.00363	-0.372	Zinc Finger MYM-type Containing 3	Ovary, Testis
**NOTCH2 NL**	1	0.009657	-0.348	Notch 2 N-terminal Like	Testis, Skin
**TIPRL**	1	0.007794	-0.34	TOR Signaling Pathway Regulator	Endometrium, Brain
**CAMK1D**	1	0.005376	-0.333	Calcium/Calmodulin dependent Protein Kinase ID	Brain, Skin
**EFNA1**	1	0.00632	-0.324	Ephrin A1	Placenta, Lung
Genes associated with male lithium non-responders					
Gene	Adjusted P-value	P-value	Log fold change	Gene description	Highest gene tissue expression
**RPS23**	1	0.013306	1.521	Ribosomal Protein S23	Ovary, Bone Marrow
**IRF2BPL**	1	0.010952	1.005	Interferon Regulatory Factor 2 Binding Protein Like	--
**HLA-C**	1	0.003461	0.997	Major Histocompatibility Complex, Class I, C	Lung, Bone Marrow
**RGPD1**	1	0.001745	0.76	RANBP2-like and GRIP Domain Containing 1	Testis, Liver
**ASGR2**	1	0.019947	0.598	Asialoglycoprotein Receptor 2	Liver, Gall Bladder
**LPAR1**	1	0.01374	0.453	Lysophosphatidic Acid Receptor 1	Brain, Placenta
**RRN3P1**	1	0.017025	0.42	RRN3 homolog, RNA Polymerase I Transcription Factor Pseudogene 1	Thyroid, Lymph Node
**TOMM34**	1	0.016655	0.416	Translocase of Outer Mitochondrial Membrane 34	Testis, Adrenal
**ACAD11**	1	0.015882	0.405	Acyl-CoA Dehydrogenase Family Member 11	Kidney, Liver
**CEBPE**	1	0.00269	0.404	CCAAT/enhancer Binding Protein Epsilon	Bone Marrow, Small Intestine
**CMIP**	1	0.017203	0.394	C-Maf Inducing Protein	Brain, Small Intestine
**IGSF6**	1	0.011786	0.38	Immunoglobulin Superfamily Member 6	Spleen, Appendix
**HDHD2**	1	0.01764	0.361	Haloacid Dehalogenase Like Hydrolase Domain Containing 2	Brain, Thyroid
**LMO4**	1	0.012872	0.359	LIM Domain Only 4	Brain, Stomach
**BACE2**	1	0.000711	0.353	Beta-site APP-Cleaving Enzyme 2	Stomach, Gall Bladder
**TPP1**	1	0.00061	0.341	Tripeptidyl Peptidase 1	Spleen, Appendix
**GALNS**	1	0.007613	0.341	Galactosamine (N-acetyl)-6-Sulfatase	Bone Marrow, Testis
**SYNM**	1	0.019042	0.322	Synemin	Esophagus, Prostate

**Table 5.** Differentially expressed genes between Female Responders and Female Non-Responders at baseline with a log fold-change threshold of 0.3.

Genes associated with female lithium responders					
Gene	Adjusted P-value	P-value	Log fold change	Gene description	Highest gene tissue expression
**FAM117B**	0.998	0.0048554	0.5257	Family with Sequence Similarity 117 Member B	Testis, Adrenal
**STAMBPL1**	0.998	0.0074433	0.39	STAM Binding Protein Like 1	Adrenal, Testis
**CD248**	0.998	0.0038199	0.3626	CD248 Molecule	--
**IFIH1**	0.998	0.0075822	0.3453	Interferon Induced with Helicase C domain 1	Spleen, Appendix
**GPR160**	0.998	0.0071723	0.3394	G Protein-coupled Receptor 160	Small Intestine, Duodenum
**STAP1**	0.998	0.0053096	0.3222	Signal Transducing Adaptor Family Member 1	Lymph Node, Appendix
**YEATS4**	0.998	0.0089003	0.3103	YEATS Domain Containing 4	Testis, Bone Marrow
**CD83**	0.998	0.0004367	0.3014	CD83 Molecule	Bone Marrow, Lymph Node
**TMOD2**	0.998	0.0081514	0.3012	Tropomodulin 2	Brain, Appendix
Genes associated with female lithium non-responders					
Gene	Adjusted P-value	P-value	Log fold change	Gene description	Highest gene tissue expression
**GOLGB1**	0.998	0.0003716	-0.6536	Golgin B1	
**RASA4CP**	0.998	0.0030349	-0.4554	RAS p21 Protein Activator 4C, Pseudogene	Spleen, Endometrium
**NACC2**	0.998	0.0061286	-0.3803	NACC Family Member 2	Brain, Fat
**EDARADD**	0.998	0.0021425	-0.3553	EDAR Associated Death Domain	Urinary Bladder, Kidney
**ZNF573**	0.998	0.0058465	-0.3463	Zinc Finger Protein 573	Thyroid, Spleen
**ALDH2**	0.998	0.0031872	-0.335	Aldehyde Dehydrogenase 2 Family (mitochondrial)	Fat, Liver
**TAPBPL**	0.998	0.0032596	-0.3206	TAP Binding Protein Like	Duodenum, Small Intestine

## Discussion

The purpose of this investigation was to define gender-specific transcriptome-level regulators of lithium treatment response prior to the initiation of lithium treatment. We first established the gender-relevant transcriptional control genes across all study-participant blood samples and specifically to male- and female-responders using a differential gene expression threshold of 0.5. We found that in the downloaded data from the Gene Expression Omnibus, some patients were mislabeled as males and females. Therefore in our first quality control analysis that established the methodology for subsequent gender-specific lithium responders, the following Decision Tree rule for accurate classifying of gender: if RPS4Y1 < 9.643, then patient is female with a probability of 100% and if RPS4Y1 ≥ 9.643, then the patient is a male with a lower probability. The differential gene expression threshold of 0.5 was found to be adequate and corroborated with similar studies that used a similar threshold for establishing gene transcription signatures ( Jansen *et al*., 2014; Mayne *et al*., 2016). However, when comparing the male-responders to male non-responders, as well as, the female responders to female non-responders, we set an inclusion fold-change threshold to 0.3. This approach is not unusual, since it is already established that both large and subtle expression changes produce to significant biological and physiological processes ( Wurmbach *et al*., 2002). Our results are hypothesis-generating and establish a computational methodology that provides insight to the importance of subgroup analysis in genomic medicine, irrespective of patient small sample-sizes. The end-goal of such analyses serves as a testing methodology for establishing gene screening panels to improve precision medicine in vulnerable and high-risk patient populations. In these patient populations, it is often not feasible to wait for weeks to determine whether a prescribed medication will work and in some cases manic patients are neither able to fully comprehend and be objectively assessed using the CGI-BP-S ( Spearing *et al*., 1997).

When reviewing the heat-map and dendrogram hierarchical cluster analysis patterns, specifically the numerous non-responders clinically-labeled and illustrated in Figure 6, they suggest that the underlying etiology resulting in clinical symptoms (e.g. mania) that led to the diagnosis of bipolar disorder may need re-classification. Further, the subsequent treatments may need to be tailored in data-driven computational psychiatry approaches. In Figure 6, for the females, the samples in the center cluster illustrates that a group of patients are clear non-responders while the patients clustered in the far-right are partial-responders, from a molecular perspective. The natural questions that arise are: (1) How to best convert the non- and partial-responders to treatment-responders? (2) Is a behavioral intervention, in this select group of patients, for whom lithium is not effective, the best answer because the symptoms maybe of a different etiology? If indeed the symptoms are of a different etiology (e.g. inflammatory), from the lithium treatment-responders, then other diagnostic (e.g. electrophysiological neuroimaging) tools may be warranted and corresponding most efficacious treatments sought.

When differentiating between male and female patients, we found that the Ribosomal Protein S4, Y-linked 1 ( *RPS4Y1*, adjusted p-value=7.36E-47) male-linked gene and the X Inactive Specific Transcript ( *XIST*, adjusted p-value=2.98E-36) female-linked gene were the most differentially expressed among genders, which is consistent with previously published studies ( Guillén *et al*., 2014; Jansen *et al*., 2014; Mayne *et al*., 2016). The genes that are specific to male lithium responders, relative to female lithium responders, are *RBPMS2, SIDT2, CDH23, LILRA5*, and *KIR2DS5*. Using the same methodology, genes identifying female lithium responders, relative to male lithium responders, are *HLA-H, RPS23, FHL3, RPL10A, NBPF14, PSTPIP2, FAM117B, CHST7*, and *ABRACL*. The Neuroblastoma Breakpoint Family Member 14 ( *NBPF14*, adjusted p-value=0.0462, Fold-change=0.586) achieved the Benjamani-Hochberg adjusted p-value of 0.0462, and has been reported to be associated with cortical neurogenesis ( Suzuki *et al*., 2017).

Computational psychiatry methods that analyze objective clinical signals (e.g. electroencephalography) and various data-types (e.g. gene expression [RNA], single-nucleotide polymorphisms [DNA], plasma drug concentrations) to classify patients in psychiatry, as advocated by the National Institute of Mental Health’s Research Domain Criteria (RDoC), are essential in psychiatry, especially in patients with developmental delay, language difficulty, and conditions of potentially different etiologies than traditionally taught ( Clark *et al*., 2017; Eugene & Masiak, 2016). Ideally, in such cases, alternative FDA-approved mood stabilizers may be initially selected prior to any pharmacological intervention by simply using a blood test. Perhaps, a gene expression screening panel at baseline, prior to the initiation of lithium and/or other FDA-approved mood stabilizer, may be better in high-risk patient populations.

These findings suggest that when implementing genomic medicine, clinical research teams should move beyond the single-gene approach when screening for treatment response biomarkers. This approach is currently the standard when screening for patient toxicity at standard doses in poor or ultra-rapid metabolizers using drug pharmacokinetics; however, as more transcription factors are discovered that regulate the cytochrome (CYP) P-450 system of genes, multi-gene pharmacokinetic panels are inevitable and may be included in future Clinical Pharmacogenetics Implementation Consortium (CPIC) guidelines. Next, medical management of patients with mania and psychosis either with pharmacotherapy and/or behavioral intervention should be tailored to biological gender due to known neuronal circuitry differences in age-matched patients with psychosis ( Eugene *et al*., 2015). Further, as a result of lithium not being hepatically metabolized, but rather transported and renally excreted as well as, the known myriad drug-drug interactions, patient dose selection may benefit from pharmacometrics modeling by American Board of Clinical Pharmaology certified physicians in applied clinical pharmacology/clinical pharmacology ( Perera *et al*., 2014; Zetin *et al*., 1986).

**Figure 5.**
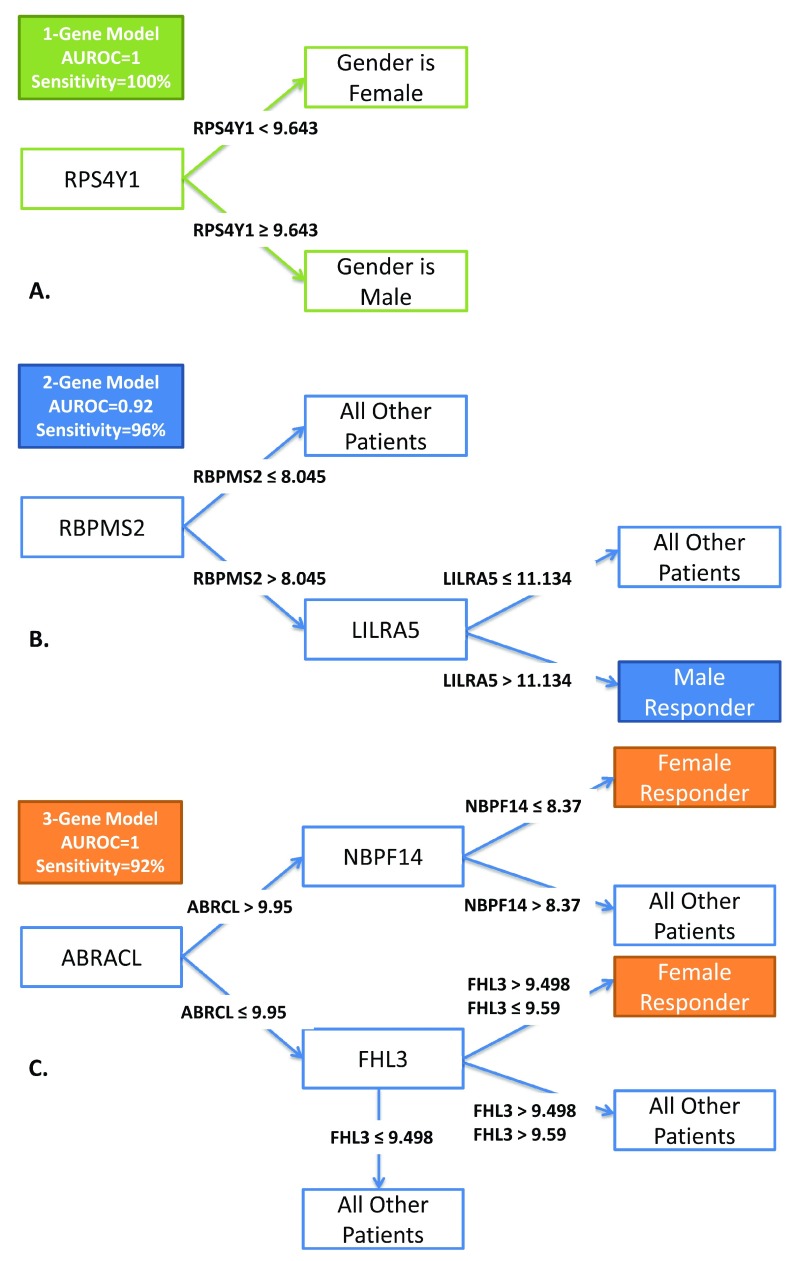
Machine learning classification results of gene expression levels that determine ( **a**) gene expression sample gender using a 1-gene (RPS4Y1) model with a sensitivity of 100% and an area under the receiver operator curve (AUROC) of 1, ( **b**) male lithium treatment responders using a 2-gene (RBPMS2 and LILRA5) model with an AUROC of 0.92, and ( **c**) female lithium treatment responders using a 3-gene (ABRACL, NBPF14, and FHL3) model with an AUROC of 1.

Further, clinical pharmacologist physicians are essential for advancing genomic medicine and providing consults in pharmacogenomics. These physicians would confirm the applicability of embedding machine learning results integrated within artificial intelligence applications in the electronic medical record. Figure 5 shows the machine learning classification results of gene expression levels that determine (a) sample gender, (b) male lithium treatment responders, and (c) female lithium treatment responders. These very study results – though with a small treatment responder population – presents an approach for data science and engineering methods for use in genomics and medicine.

**Figure 6.**
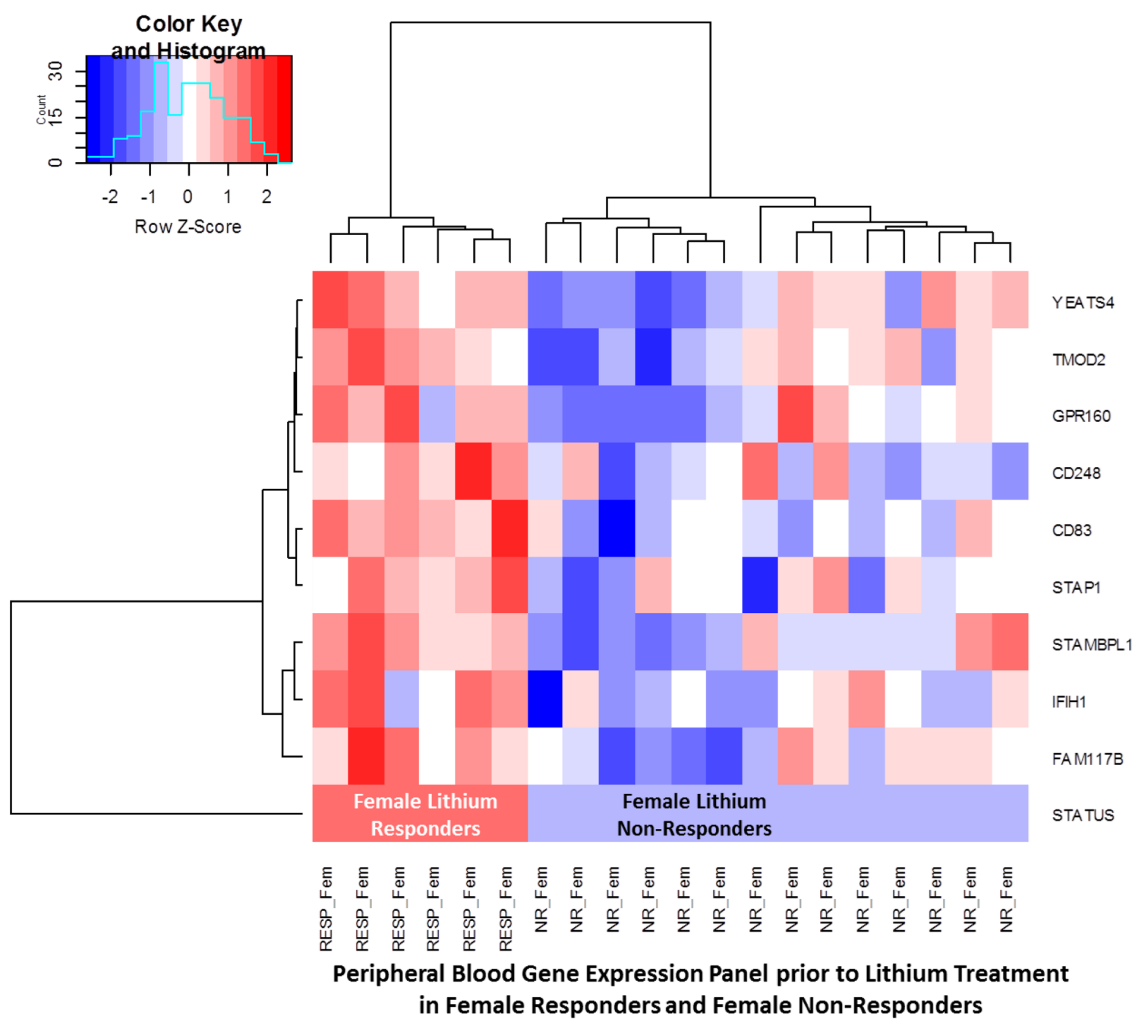
Heat-map and dendrogram overview of the two-way unsupervised hierarchical cluster analysis of differentially expressed genes prior to lithium treatment in female lithium responders (n=6, RESP_Fem) and female non-responders (n=14, NR_Fem).

The limitations of our analysis – as in most pharmacogenomic clinical studies – are understandably due to a small patient sample size and multiple-comparison p-value adjustments ( Dudoit *et al*., 2003). The fundamental aims of our research questions were designed to answer biological questions of gender and clinical response to lithium and not meant to be driven exclusively by multiple comparisons adjusted p-values or limited by not having enough patients. This approach has led to various successes in pharmacogenomics, specifically, in genome-wide association studies; however, understandably, the limitations are thoroughly acknowledged. In reference to patient sample sizes, 9 out of the 28 patients who received lithium and optimal therapy were classified as lithium treatment responders. Further, 30% of men and 33% of women, who were treated with lithium, were found to be responders at the respective gender categories ( Beech *et al*., 2014). However, the strengths of our findings are in the gender-gene screening capability for lithium treatment-responders in the general population of 60 patients at baseline, minus the tested responder group. Opportunities exist for any further clinical studies, prospective clinical trials, and application of the methods outlined in this work for other therapeutic agents across several medical specialties and other disciplines.

## Conclusion

We explored the Lithium Treatment-Moderate dose Use Study clinical trial gene expression data with the aim of identifying gender-specific transcriptome-level regulators of lithium treatment response. We found that male and female labeled patients were misclassified and used the following Decision Tree rule for accurate classifying of gender: if RPS4Y1 < 9.643, then patient is female with a probability of 100%. Further, using machine learning, we successfully developed a pre-treatment gender- and gene-expression-specific predictive model selective for lithium responders with an AUROC of 0.92 for male lithium responders (sensitivity=96%) and an AUROC of 1 for female lithium responders (sensitivity=92%). Moreover, by using well-established Bayesian statistical methods, to identify differentially expressed genes and then machine learning, we discovered 2-genes (RBPMS2 and LILRA5) selective for male lithium responders and 3-genes (ABRACL, FHL3, and NBPF14) selective for female lithium responders that will inform physicians and the medical staff of whether the patient will respond to lithium prior to being prescribed the mood stabilizer. Further, due to the small number of patients classified as responders from the clinical trial, our results should be confirmed. Lastly, in an overall context, our results suggest that the methodology used in this analysis may be extended to other therapeutic drug classes and provides insight to the gender-based gene transcriptome differences influencing lithium pharmacodynamics.

## Data availability

Data used in this study are available from https://www.ncbi.nlm.nih.gov/geo/query/acc.cgi?acc=GSE45484
