# Supplementary material for: Predicting lithium treatment response in bipolar patients using gender-specific gene expression biomarkers and machine learning
Source: F1000Res. 2018 Dec 7;7:474. Originally published 2018 Apr 18. [Version 3] doi: 10.12688/f1000research.14451.3 (PMC6381805; doi:10.12688/f1000research.14451.3)
Supplement: Supplementary file 1 [file f1000research-7-18909-s0001.tgz › caf2ad95-c800-40fc-be2f-6c4d67dc41ac_updated_-_Supplementary_methods_for_Lithium_treatment_response_article.docx]

**Supplementary File 1: Supplementary methods.**

**Random Forest Model Decision Tree Rules for Classifying Male Lithium Responders using the following 2 Genes RBPMS2 and LILRAS5**

Tree 1 Rule 1 Node 2 Decision All_Other_Patients

 1: RBPMS2 <= 8.04447

-----------------------------------------------------------------

Tree 1 Rule 2 Node 4 Decision All_Other_Patients

 1: RBPMS2 > 8.04447

2: LILRA5 <= 11.13405

-----------------------------------------------------------------

Tree 1 Rule 3 Node 5 Decision Male_Responder

 1: RBPMS2 > 8.04447

2: LILRA5 > 11.13405

**Random Forest Model Decision Tree Rules for Classifying Female Lithium Responders using the following 3 Genes ABRACL, FHL3, and NBPF14**

Tree 1 Rule 1 Node 4 Decision All_Other_Patient

1: ABRACL <= 9.95025

2: FHL3 <= 9.497515

-----------------------------------------------------------------

Tree 1 Rule 2 Node 8 Decision Female_Responder

1: ABRACL <= 9.95025

2: FHL3 > 9.497515

3: FHL3 <= 9.590545

-----------------------------------------------------------------

Tree 1 Rule 3 Node 9 Decision All_Other_Patient

1: ABRACL <= 9.95025

2: FHL3 > 9.497515

3: FHL3 > 9.590545

-----------------------------------------------------------------

Tree 1 Rule 4 Node 6 Decision Female_Responder

1: ABRACL > 9.95025

2: NBPF14 <= 8.37315

-----------------------------------------------------------------

Tree 1 Rule 5 Node 7 Decision All_Other_Patient

1: ABRACL > 9.95025

2: NBPF14 > 8.37315

**Decision Tree Model Rules for Classifying Males versus Females using 1 Gene RPS4Y1**

Rule number: 3 [GENDER=Female cover=50 (69%) prob=1.00]

RPS4Y1< 9.643

-----------------------------------------------------------------

Rule number: 2 [GENDER=Male cover=22 (31%) prob=0.09]

RPS4Y1>=9.643


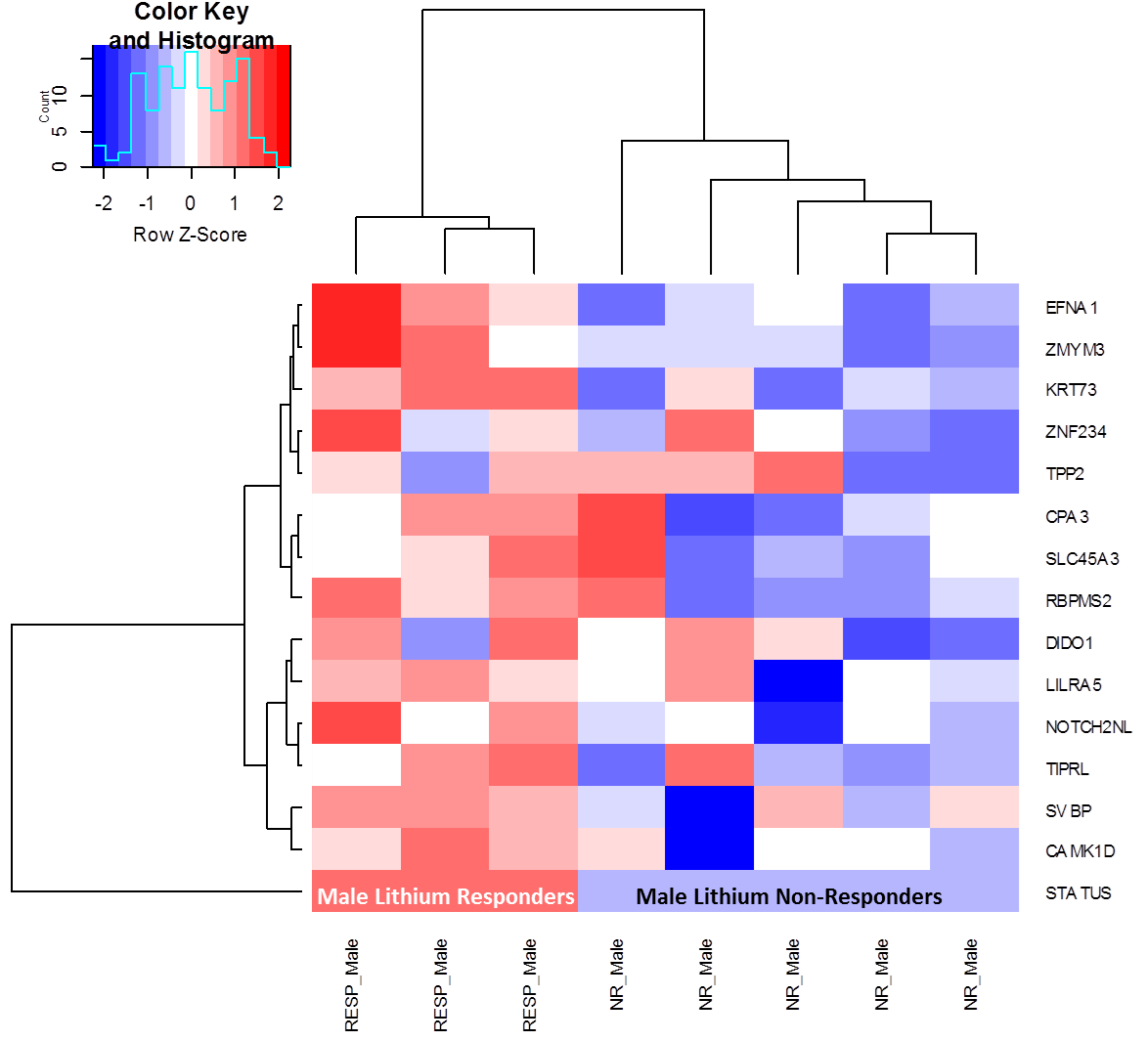


**Supplementary Figure 1: Heat-map and dendrogram overview of the two-way unsupervised hierarchical cluster analysis of differentially expressed genes prior to lithium treatment in male lithium responders (n=3, RESP_Male) and male lithium non-responders (n=7, NR_Male).**
