# Supplementary material for: Predicting lithium treatment response in bipolar patients using gender-specific gene expression biomarkers and machine learning
Source: F1000Res. 2018 Dec 7;7:474. Originally published 2018 Apr 18. [Version 3] doi: 10.12688/f1000research.14451.3 (PMC6381805; doi:10.12688/f1000research.14451.3)
Supplement: Peer review history

# Peer review: Reviewer response for version 3

**DOI:** 10.5256/f1000research.18909.r43262

**Published:** 2019-02-18

**Authors:** Duan Liu

**Affiliations:** 1 Division of Clinical Pharmacology, Department of Molecular Pharmacology and Experimental Therapeutics, Mayo Clinic, Rochester, MN, USA

Using a published data set, the authors built a model based on gender-specific gene expression to predict lithium treatment response in bipolar disorder patients. Here are my questions and concerns:

1. The authors realized that the sample size is small and the results will need further confirm. Is there any other similar (lithium treatment response) clinical trial/data available to test the predicting model?
2. After the Step 1 analysis, why not exclude the four samples that their genders had been mis-classified? (Since the clinical data for those four patients may be also mis-labelled.) Data accuracy is critical for building models, especially when dealing with small sample size.
3. Those three genes, the *ABRACL*, *FHL3*, and *NBPF14*, which classified female responders seem not differentially expressed between female responders and female non-responders (Table 5). Please explain why this might be happened.
4. What is the known function of those 5 genes that chosen for classification of male and female responders? The authors might want to discuss the possible molecular mechanism of those gene function related to lithium treatment response in bipolar disorders.
5. Since the authors re-analyzed data from a previously published study, a comparison of the findings from this study to the original one may be necessary. I understood that the original study looked at differences between responder and non-responders, regardless of genders. Is there any gene, of which its expression differentiates responder and non-responder, overlapped with what the authors found in this study? What will be found if apply a machine learning approach to this data set without classifying patients by genders?

I have read this submission. I believe that I have an appropriate level of expertise to confirm that it is of an acceptable scientific standard, however I have significant reservations, as outlined above.

# Peer review: Reviewer response for version 3

**DOI:** 10.5256/f1000research.18909.r42767

**Published:** 2019-01-09

**Authors:** Sunil V. Kalmady

**Affiliations:** 1 Department of Psychiatry, University of Alberta, Alberta, Canada; 2 Alberta Machine Intelligence Institute, Alberta, Canada

Authors have made improvements to the manuscript in terms of clarity of methodology. Hence, now I can comment on their methods.

 Biostatistical methods are mostly sound.

 However, machine learning part of study seems have major issues:

1. Authors haven’t specified number of responders in their test sets or whether split is class-balanced. But based on their description of 70 – 30 train -test split, sample size is severely limiting for model evaluation, with mere one or two examples of positive class (responders) in test set (30% of 3 male ~ 1 ; 30% of 6 female ~ 2). As a fellow researcher, I completely respect the motivation and effort behind the efforts here, but we as scientific community should understand that the real danger of generalizing observations based on handful of cases is not so much of being underpowered to detect real effect, but of generating false positives results that add to prevailing burden of irreproducible results.
2. It seems that features (250 control gene selection, 2-gene model, 3-gene model etc..) were selected using analyses of both training and testing data partitions. This is called double-dipping and leads to invalid or over-optimistic estimates of model performance.

While above two points are deal breakers, I will also mention following points for sake of completion.

1. Hyper-parameters should also be selected ‘ *in fold*’ or their choice should be explained.
2. Baseline performance (chance level accuracy) is rather high due to class imbalance – eg: 25/28 = 89% for male responders. Reporting the confusion matrix will be more useful than sensitivity, AUC etc in such cases.
3. For small samples, consider simple linear models than complex non-linear ones such as random forest to avoid over-fitting. Also, consider leave-one-out or k-fold cross validation instead of single test-train split for better estimate of performance.

Hence, in my humble opinion, the manuscript in its current form doesn’t meet the necessary scientific rigor. That is at least without a major revision in machine learning methods, such as learning models to predict treatment response in larger undivided dataset of 60 subjects, appropriate use of feature selection etc.

I have read this submission. I believe that I have an appropriate level of expertise to confirm that it is of an acceptable scientific standard, however I have significant reservations, as outlined above.

# Peer review: Reviewer response for version 2

**DOI:** 10.5256/f1000research.16407.r39873

**Published:** 2018-11-12

**Authors:** Sunil V. Kalmady

**Affiliations:** 1 Department of Psychiatry, University of Alberta, Alberta, Canada; 2 Alberta Machine Intelligence Institute, Alberta, Canada

Eugene et al. tackles the hard question on the transcriptome predictors of clinical outcome in lithium treatment of Bipolar disorder. This research question is of clinical relevance and importance, however, there might be some issues with how the data was analyzed and presented in the manuscript. 

- The study design and methods do not seem to be coherent to a single unifying goal. Whether the goal is to predict the responder using the 'gender' and 'transcriptome' data as features? If so, such a model can be learned using standard machine learning methods. On the other hand, whether the goal is to identify are genes with statistically significant group differences in their expression? If so, this can be achieved by biostatistical inference tests of association. 

Please note that task of 'association' and 'prediction' are quite distinct in their formulation and desired objective. Authors have to be really careful about that they trying to test and claim while using both of approaches in conjunction. 

- Study design is a bit unconventional, and hence needs to be motivated and explained better. For example: Performing successive sub-group analyses partitioned on factors like gender have less power, and should be generally restricted to post-hoc tests. Why not simply use standard biostatistical tools such as factorial ANOVA with 'sex' and 'response' as between-subjects factors of interest?

I agree with Reviewer #1 that flowchart of analysis pipeline will help the understanding. Steps of sub-group selection and variable/feature selection can be indicated in this flowchart. Care should be taken to avoid the circularity that can arise from selection because statistical inference can be invalid whenever the results statistics are not inherently independent of the selection criteria under the null hypothesis. 

- Authors might be asking too many questions with limited data in hand. Sample size might not enough to study individual effects of multiple factors - such as treatment-type, response and then, the gender. Cell-wise sample sizes resulting from this 8-way split is less than 10 for all but two cells (less than 5 for 3 cells). Suitability of applied statistical tests and generalizability of their claims are questionable here. Authors should also think about 1:2 skew in male:female ratio, which makes this issue worse. Study can greatly benefit from asking specific and limited hypothesized questions. 

Also, since multiple objectives are stated, methods and results section can describe each objective separately for sake of better clarity.  

   

- Machine learning methods are not described. The methods used for learning of model and its evaluation process needs be specified. Example: How was training and test splits performed? How was feature selection performed? How were the hyper-parameters optimized? Whether the reported performance metrics are for training or testing sets? Whether the discovery dataset used for identifying '250 genes' disjoint from validation set? etc. Without these details, it is hard to comment on validity of a predictive study.

I have read this submission. I believe that I have an appropriate level of expertise to confirm that it is of an acceptable scientific standard, however I have significant reservations, as outlined above.

# Response

**Published:** 2018-11-27

**Authors:** Andy Eugene

**Affiliations:** Independent Researcher, USA

**Please see our responses in bold-face to the comments from Referee #2. We thank you for reviewing this article.**

 Eugene et al. tackles the hard question on the transcriptome predictors of clinical outcome in lithium treatment of Bipolar disorder. This research question is of clinical relevance and importance, however, there might be some issues with how the data was analyzed and presented in the manuscript. 

- The study design and methods do not seem to be coherent to a single unifying goal. Whether the goal is to predict the responder using the 'gender' and 'transcriptome' data as features? If so, such a model can be learned using standard machine learning methods. On the other hand, whether the goal is to identify are genes with statistically significant group differences in their expression? If so, this can be achieved by biostatistical inference tests of association. 
- Response: **First and foremost, I sincerely appreciate you taking time out to review this article and provide constructive feedback. In answering these points, this paper addressed questions that were not addressed in the original clinical study and attempted not to duplicate work previously published. Hence, is why the methods are creative and not employ standard biostatistical inference tests of association. For example, we aimed to test the hypothesis if gender influenced selecting transcriptome signatures associated with lithium efficacy. Therefore, in order to accomplish this task, we conducted a quality control test that identifies known gender-biased transcriptome signatures. This proved to be essential and is why we identified that several patients were misclassified as male/female. Results are shown, documented, and corroborated with other studies identifying the same genes as being gender-specific. Next, standard statistical tests were indeed used, however, we chose to use those adapted for Gene Expression analysis as has been developed using the “limma” package and for machine learning methods, we used the Random Forest algorithm. We then aimed to develop a predictive model to see if this may be a first-step for other medical research teams to validate with further clinical studies and for Clinical Pharmacology laboratories to pursue and develop novel experiments to determine lithium’s effect in cells, tissues, laboratory animals, and later in humans. **

 

 Please note that task of 'association' and 'prediction' are quite distinct in their formulation and desired objective. Authors have to be really careful about that they trying to test and claim while using both of approaches in conjunction. 

Response: **Well said and this is duly noted. We first aimed to identify genes associated with responders and hoped to identify if using those genes would help in creating hypothesis-generating predictors treatment response, only to be later validated by other studies. Thank you for the comment.**

- Study design is a bit unconventional, and hence needs to be motivated and explained better. For example: Performing successive sub-group analyses partitioned on factors like gender have less power, and should be generally restricted to post-hoc tests. Why not simply use standard biostatistical tools such as factorial ANOVA with 'sex' and 'response' as between-subjects factors of interest?
- Response: **While your point with “gender” should be restricted to post-hoc analysis, we are looking to make gender the primary point of our analysis in patients responding to lithium treatment and specifically not repeat the analysis from the original publication in Nature. This was attempted on the original article, however, we clearly identified that there was patient-gender misclassification in the original study. So, we sought another route of analysis to ensure that gender was a primary point of analysis and not side-lined to the post-hoc analyses. However, we do understand that this compromises statistical power and therefore, sought not to analyze the data with ANOVA, because we are addressing minor gene expression level changes that might have real-world clinical insight. This is a hypothesis-generating analysis, however, we do thank you as well for this comment.**

I agree with Reviewer #1 that flowchart of analysis pipeline will help the understanding. Steps of sub-group selection and variable/feature selection can be indicated in this flowchart. Care should be taken to avoid the circularity that can arise from selection because statistical inference can be invalid whenever the results statistics are not inherently independent of the selection criteria under the null hypothesis. 

Response: **We appreciate the request for having a graphical flowchart depicting the analysis pipeline and have included the figure in the updated version of the manuscript. We are confident that circularity is not an issue, given the specific aims of our analysis. These methods are determined to seeking to identify the influence of linked gender-drug-response to genes at baseline and not after treating with the mood stabilizer. The gender differences in clinical practice are a well-documented reality and we literally sought to identify any signal, on the gene expression level, to address the clinical question rather than entirely use traditional statistical methods which did not necessarily translate to clinical translation. With our results, we are expecting laboratories having strengths in gene knock-down/out and gene over-expression experiments to identify the mechanisms to lithium’s efficacy. This is an old drug and until this day, most textbooks lack knowledge of the drug’s mechanism. These mechanisms may be attributable to biological, biochemical, gene expression, hormonal, and proteomic differences that we are aiming to identify here in this article. Please see the new figure showing the data analysis pipeline used in this paper.**

- Authors might be asking too many questions with limited data in hand. Sample size might not enough to study individual effects of multiple factors - such as treatment-type, response and then, the gender. Cell-wise sample sizes resulting from this 8-way split is less than 10 for all but two cells (less than 5 for 3 cells). Suitability of applied statistical tests and generalizability of their claims are questionable here. Authors should also think about 1:2 skew in male:female ratio, which makes this issue worse. Study can greatly benefit from asking specific and limited hypothesized questions. 
- Response: **We do appreciate your robustness in identifying the obvious study limitations due to sample-size, however, we are limited to the feasibility, cost of research, patient population, and all of the work accomplished from the original study team stemming from Case Western Reserve University, Massachusetts General Hospital (Harvard University), Stanford University, Yale University, the University of Pittsburgh, Texas Health Science Center at San Antonio, and the University of Pennsylvania that uploaded this data into the Gene Expression Omnibus (GEO) database maintained by the National Institutes of Health. This was a massive undertaking in a multi-site trial. The sample-size limitations are classically used to not have results generalized, however, we ask you realize that this work not necessarily straight-forward to accomplish with in the real-world of medical care in mental health. Nevertheless, your points are well noted. **
- Response: **The 1:2 male:female skew you are referring to is exactly what is seen in clinical medicine. Females tend to respond more so than males and I clearly stated this in the introduction of the paper. We are working with the data that has been uploaded and have not found any other datasets in GEO. However, please understand that our efforts are indeed, as you clearly pointed in the beginning of this review, that this is a difficult clinical question to answer. Rather than saying we do not have enough samples, we aimed to do ‘something’ rather than let the dataset sit in GEO while patients are in need and laboratories have the capability and funding to seek follow-up studies. We appreciate your clear expertise and concern. However, we are working to create hypothesis-generating results to be later confirmed, expanded-upon, and validated for sick patients. Therefore, the generalizability of our claims are clearly limited to the dataset we obtained from clinical study of the aforementioned university hospitals. Of the 60 (sixty) patients treated with Lithium, literally only 9 responded in follow-up my expert medical teams and high quality care. Hence the need to find ‘some signal’ with the data at-hand in the form of expression patterns of lithium treatment responders. Thank you for the statement and again these are well-noted points you stated here.**

 

 Also, since multiple objectives are stated, methods and results section can describe each objective separately for sake of better clarity.  

   

- Machine learning methods are not described. The methods used for learning of model and its evaluation process needs be specified. Example: How was training and test splits performed? How was feature selection performed? How were the hyper-parameters optimized? Whether the reported performance metrics are for training or testing sets? Whether the discovery dataset used for identifying '250 genes' disjoint from validation set? etc. Without these details, it is hard to comment on validity of a predictive study.
- Response: **Thank you for these questions and comments. We have updated the methods to better explain the approach used in the study. The new graphical analysis pipeline will help in explaining the approach. We also added the final Decision Tree diagrams to identify male- and female-treatment responders. Thank you for the review and we have made considerable updates to this version of the paper to address these concerns and improve this research manuscript.**

# Peer review: Reviewer response for version 2

**DOI:** 10.5256/f1000research.16407.r34437

**Published:** 2018-06-27

**Authors:** Ming-Fen Ho

**Affiliations:** 1 Division of Clinical Pharmacology, Department of Molecular Pharmacology and Experimental Therapeutics, Mayo Clinic, Rochester, MN, USA

I have read this submission. I believe that I have an appropriate level of expertise to confirm that it is of an acceptable scientific standard.

# Peer review: Reviewer response for version 1

**DOI:** 10.5256/f1000research.15730.r33941

**Published:** 2018-05-21

**Authors:** Ming-Fen Ho

**Affiliations:** 1 Division of Clinical Pharmacology, Department of Molecular Pharmacology and Experimental Therapeutics, Mayo Clinic, Rochester, MN, USA

The authors demonstrated that sex-differences gene expression might contribute to lithium treatment response using microarray expression data.

 Major comments:

1. A samples size of 60 might be too small to determine the sex effects. Can the sample size n=60 provide adequate power for data interpretation, especially separated men and women for study sex-effect on gene expression?
2. The authors stated that their predictive model for lithium responders with an ROC AUC 0.92 for men, and 1 for women.  If the prediction accuracy is so significant, what are the potential biological mechanisms beyond these genes? More discussion regarding the biology of those genes should be included in the paper. Once again, if the prediction accuracy is so significant, it is needed a replication study using different data sets? In summary, the authors claimed the prediction model with very high accuracy; it should be included either functional validation of those genes or a replication study population.

 

 Specific comments:

1. Methods - study design, it might be better to use a flow chart to demonstrate the study design.
2. Methods - study design, please clarify the rationale of filtering out “250” genes.
3. Table 1 shows total study population n=60, but figure 1 legend shows male: n=41, female: n=39? 
4. Figure 2:  please elaborate the data presented in Figure 2. The key results for each of the four panels should be summarized in Results.
5. Table 2 and Table 4, the log FC threshold of 0.5 or 0.3 might be too low. The changes in gene expression are very subtle in Table 4.
6. Table 2, are there any gene up-regulated in males? /downregulated in females?
7. Limitations of the study should be addressed in Discussion.

I have read this submission. I believe that I have an appropriate level of expertise to confirm that it is of an acceptable scientific standard, however I have significant reservations, as outlined above.

# Response

**Published:** 2018-05-21

**Authors:** Andy Eugene

**Affiliations:** Independent Researcher, USA

**Major Comment Responses:**

**Response 1:**

This point is well noted; however, it is important to realize that our gender-effects of gene expression is consistent with other studies noted within the paper and shown below:

Jansen, Rick, et al. "Sex differences in the human peripheral blood transcriptome." *BMC genomics* 15.1 (2014): 33.

Mayne, Benjamin T., et al. "Large scale gene expression meta-analysis reveals tissue-specific, sex-biased gene expression in humans." *Frontiers in genetics* 7 (2016): 183.

Further, our gender-specific results met the Benjamini-Hochberg multiple comparisons criteria adjustment due to multiple comparisons.

**Comment Response 2:**

We welcome and thank the reviewer’s comments on the biological mechanisms beyond these genes. Clearly, it is well noted and cited in the paper that in clinical practice there is a wide inter-individual variability in the treatment and response to treatments of biplolar disorder. Moreover, these patients were not treated with lithium monotherapy, alone, and therefore further insight into the biological mechanisms were left out due to these patients were treated with an “Optimal Therapy” that includes a variety of other FDA-approved mood stabilizers.

In reference to the comment regarding the prediction accuracy, we agree that the study may warrant functional validation in a laboratory; however, it is beyond the scope of our computational psychiatry study and we will leave the functional genomics characterization of the genes to investigators seeking to pursue the findings from our results.

# Response

**Published:** 2018-05-21

**Authors:** Andy Eugene

**Affiliations:** Independent Researcher, USA

**Specific Comment Responses:**

 We thank you for your specific comments and have addressed several of the pertinent points in your review. For all differentially expressed results reported throughout tables within the manuscript, we changed the wording from genes up-regulated or down-regulated in males or females to a clearer description statement that of genes-associated with males or females. However, we thought not necessary to include an extra figure, but rather encourage the reader to (1) review the study design section within the methods to better understand the computational approach used in our analysis and (2) read the systematic tabular reporting of the results in the manuscript text as well to understand that study approach.

 For the caption in Figure 1, we thank you for the comment and have updated the sample sizes for males and female patients. The updated Figure 1 text reads: Males (n=20; with 40 pre- and post-treatment samples) and Females (n=40; with 80 pre- and post-treatment samples).

 The comments regarding: (1) the fold-change of 0.5 and 0.3 being subtle and (2) the study limitations, are already specifically addressed within the original version of the manuscript. Again, it is well established and referenced within the text that small changes in gene expression have already been reported to result in major functional outcomes in human physiology.

 We will update the variable importance illustration shown in Figure 2 and that will be added to the updated version of the manuscript.
